# Dynamics of laser-driven proton acceleration exhibited by measured laser absorptivity and reflectivity

**DOI:** 10.1038/srep43548

**Published:** 2017-03-08

**Authors:** J. H. Bin, K. Allinger, K. Khrennikov, S. Karsch, P. R. Bolton, J. Schreiber

**Affiliations:** 1 Fakultät für Physik, Ludwig-Maximilians-Universität München, D-85748 Garching, Germany; 2 Max-Planck-Institut für Quantenoptik, D-85748 Garching, Germany

## Abstract

Proton acceleration from nanometer thin foils with intense laser pulses is investigated experimentally. We analyzed the laser absorptivity by parallel monitoring of laser transmissivity and reflectivity with different laser intensities when moving the targets along the laser axis. A direct correlation between laser absorptivity and maximum proton energy is observed. Experimental results are interpreted in analytical estimation, exhibiting a coexistence of plasma expansion and light-sail form of radiation pressure acceleration (RPA-LS) mechanisms during the entire proton acceleration process based on the measured laser absorptivity and reflectivity.

Plasma expansion driven by irradiation of targets by intense laser pulses has attracted scientific interest for decades; especially with laser intensities beyond the threshold for heating electrons to relativistic energies (>1.37 × 10^18^ W/cm^2^)^1^. Expansion velocities of at least ten percent of the speed of light, yield tens of MeV ion kinetic energies (per nucleon)^2^. For thin target foils of micrometer or even sub-micrometer thickness ion emission attributed to target normal sheath acceleration (TNSA) has been observed predominantly in directions perpendicular to the original upstream (irradiated) and downstream (nonirradiated) target surfaces^3^,^4^. Highest ion velocities have been reported for targets of thickness less than a few micrometers and commonly limited by premature destruction of the target due to pre-pulses^5^. Ion energy spectra obtained with TNSA are observed to be characteristically broad with an exponentially decaying amplitude towards a maximum or cut-off high energy. As an indicator of laser-ion acceleration performance, numerous theoretical models and simulations have addressed this cut-off (see, for example^6^,^7^,^8^, and references therein). Of the many laser parameters influencing ion energy^5^,^9^,^10^,^11^,^12^, the efficiency with which plasma electrons are heated by absorbing laser photons *η*_*L*_ is the most critical. The subsequent ‘hot’ electrons establish the electric field at the target surface which then drives ion acceleration. We refer to the TNSA mechanism simply as plasma expansion.

A different ion acceleration mechanism, the light sail form of radiation pressure acceleration (RPA-LS) has also been the subject of renewed interest^13^,^14^,^15^,^16^,^17^,^18^. First indications of this efficient and hence attractive mechanism have been observed in experiments^19^,^20^,^21^. According to this scheme, a small neutral object of sufficiently low mass gains momentum and kinetic energy due to the reflection of impinging laser light that is also Doppler-shifted (to lower (redder) frequencies). This nanoscopic interplay within the small target is still based on a charge separation, i.e. the laser ponderomotively pushes the electrons forward (the sail), pulling the ions (the cargo) along via the electric field between them. The conditions under which RPA should be observable are the subject of theoretical^18^,^22^ and experimental^23^ studies. It is clear though, that to maximize the velocity of the object via RPA (i.e. the velocity of the ions and electrons), the reflectivity *r*_*L*_ of the plasma to the laser light (i.e. the ratio of number of photons reflected from the target to the number of incident photons) must also be maximized and approach unity. Therefore in this ideal case for which there is no absorptivity (*η*_*L*_ = 0), laser pulse energy would not be converted into internal energy within the object and the plasma would not expand.

In reality neither the reflectivity nor the absorptivity reach the desired optimum values and one seeks a realistic optimization. Reflectivity *r*_*L*_ and absorptivity *η*_*L*_ depend on an assortment of laser and plasma parameters, some of which can be relatively inaccessible thus complicating theoretical prediction. Here we provide experimental results from direct measurements of laser reflectivity *r*_*L*_ and transmissivity *t*_*L*_ over a range of laser intensities. In this work normally incident focused laser pulses irradiate nanometer thickness diamond-like carbon (DLC) foils^24^ that include about 10% hydrogen impurity. By directly measuring laser pulse reflectivity and transmissivity we also observe a clear correlation between laser absorptivity and proton acceleration. The experimental data are inline with a simple analytical model that indicates the coexistence of plasma expansion and RPA-LS mechanisms in proton acceleration.

## Results

**Figure 1:**
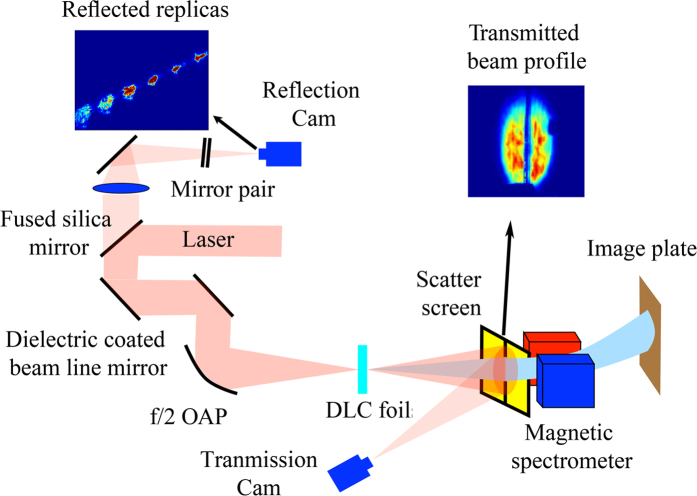
Schematic of experimental setup. A 30 fs laser pulse focused by a 90° off-axis-parabola (OAP) irradiates the target (DLC foils) at normal incidence. The energy and angular distribution of the protons along the direction of the laser is measured by a magnetic spectrometer. The reflected and transmitted laser pulses are measured simultaneously. The right inset presents a measured transmitted image in vacuum (no target) for calibration and the left inset shows a typical reflected image of multiple replicas. The replicas are obtained from a Fabry-Perot etalon placed in front of the reflectivity camera, to ensure that at least one well-illuminated spot can be analysed for each laser shot.

In the experiment, the reflectivity *r*_*L*_ and transmissivity *t*_*L*_ of the incident laser lights were quantitatively determined via charged coupled devices (CCD) as detectors. The detailed schematic of the experimental setup is shown in Fig. 1. The transmissivity *t*_*L*_ is obtained by integrating the transmitted laser profile from a scatter screen located in front of the magnetic spectrometer. The reflected laser light was recollected over the full aperture via the off-axis-parabolic mirror and refocused through a Fabry-Perot etalon to multiple spots. The reflectivity is obtained by choosing one well-illuminated and non-saturated replica of the beam and integrating over this profile. Along with filters, the optical collecting system guarantees a sufficient discrimination of the primary laser light which we are interested in, from secondary radiation such as harmonics. For example, the detection sensitivity of the fundamental laser is 6 orders of magnitudes higher than 2ω harmonic. (see Method). The proton energy spectrum along the laser direction is measured via a magnetic spectrometer. In the experiments, DLC-foils with thickness of 5, 10, and 20 nm were used as targets. The position of the DLC-foils was shifted along the propagation direction of the laser, leading to a variation of the spatial laser intensity distribution (and therefore integrated fluence) on target which in turn influences the measured values of the reflectivity, *r*_*L*_ and the transmissivity, *t*_*L*_. We have not observed scattered light in other directions and can therefore define the absorptivity simply as *η*_*L*_ = 1 − *r*_*L*_ − *t*_*L*_.

**Figure 2:**
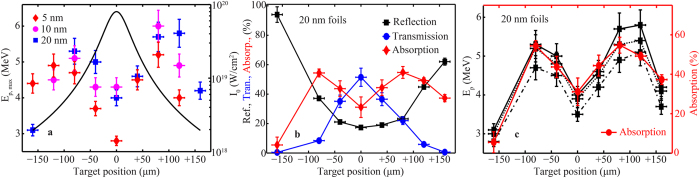
(**a**) Maximum proton energy *E*_*p,max*_ as a function of target position, where +/− means the foils were placed upstream/downstream of the laser focal plane. Different colors denote different target thickness, 5 nm (red), 10 nm (magenta), and 20 nm (blue). Regardless the target thickness, the highest energies of protons are preferably attained when the target is located about ±100 *μm* from the focal plane, where laser intensity is significantly lower than the peak values in the focal plane (8 × 10^19^ W/cm^2^). The longitudinal variation of on-target laser intensity *I*_0_ for varying target positions is indicated by the solid black curve. As indicated, when moving the target out of focal plane of ±100 µm, the laser intensity drops to 5 × 10^18^ W/cm^2^. (**b**) Measured reflectivity *r*_*L*_ (black), transmissivity *t*_*L*_ (blue) and determined absorptivity *η*_*L*_ = 1 − *r*_*L*_ − *t*_*L*_ (red) as they vary with target position for 20 nm thick DLC foils. (**c**) Thus determined absorptivity (red) and proton iso-amplitude (particle numbers) lines at equal proton yield (black curves) as a function of target position for 20 nm thick DLC foils. The black curves therefore illustrate the spatial and energy variation of a constant proton spectral amplitude, i.e., constant *dN*/*dE* = 10^4^/MeV (solid), *dN*/*dE* = 10^6^/MeV (dashed), *dN*/*dE* = 10^8^/MeV (dotted).

We exclusively observed protons with monotonically decaying energy spectra up to a well defined maximum kinetic energy. The variation of this maximum value with target position for three different target thicknesses is plotted in Fig. 2(a). Here, 0 *μm* corresponds to the target being positioned into the vaccum position of the laser focal plane (best focus). The vertical error bars indicate the uncertainty in determining the maximum proton energy. The maximum proton energy is seen to vary almost symmetrically about the focal plane. The reduced maximum proton energy in the focal plane is attributed to the temporal contrast of the laser pulse. Regardless of the initial target thickness, the highest maximum energies (up to 6 MeV) are obtained about 100 µm from the focal plane on either side which is well-beyond the 25 µm Rayleigh range. In the vicinity of these ±100 µm locations the laser pulse intensity is near 5 × 10^18^ W/cm^2^, slightly above the threshold for accelerating plasma electrons to relativistic energies. Also, it is important to note that although carbon ions compose the majority of the DLC foils, in the experiment we did not observe any carbon signals in the detectable range (above 43 MeV) of our spectrometer.

Figure 2(b) illustrates the measured values of *r*_*L*_, *t*_*L*_, along with *η*_*L*_ = 1 − *r*_*L*_ − *t*_*L*_ for 20 nm thick foils, also revealing the longitudinal symmetry. In fact, thinner targets (5 and 10 nm) behaved similarly, suggesting that the initial foil thickness plays a minor role in the observed general trend, while influencing slightly the absolute values. The longitudinal variation of *η*_*L*_ remarkably resembles the trend observed for the maximum proton energy in Fig. 2(a). Clearly, the maximum proton energy is directly correlated to the laser absorptivity *η*_*L*_. In fact, the same correlation is also observed at each constant proton spectral amplitude plotted in Fig. 2(c) in which the shape of the proton iso-amplitude lines, i.e. lines that connect the spectra at equal proton number values over the target position for 20 nm thick foils are presented. In other words, we extracted corresponding values of proton energies at specific particle numbers per MeV from the monotonically decayed spectra measured for every shot. Hence, the solid black curve here corresponding to the maximum proton energies in Fig. 2(a) is representative for the dependence of proton acceleration on absorptivity.

## Discussion

**Figure 3:**
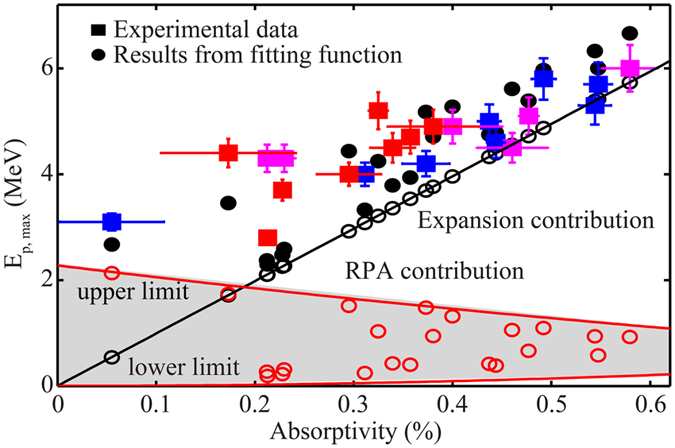
Measured maximum proton energy *E*_*p,max*_ (squares) plotted as a function of laser absorptivity *η*_*L*_. Here, different colors denote different target thickness, i.e., 5 nm (red), 10 nm (magenta), and 20 nm (blue). The black solid circles show the best fitted results by Eq. 1, including both contributions of expansion (empty black circles) and RPA-LS (empty red circles). The solid black line indicates the contribution from plasma expansion alone, showing a characteristic linear dependence *E*_*p*_ ∝ *η*_*L*_. The solid red lines marked two scaling limits of the RPA-LS mechanism, where the upper limit imposes a linearly decreasing scaling according to *E*_*rpa*_ ∝ (2 − *η*_*L*_)^2^ when *t*_*L*_ = 0, and the lower limit imposes a linearly increasing scaling according to 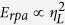 when *r*_*L*_ = 0.

In the following we aim to give a simple, analytical explanation for the above described correlation between proton energy and absorptivity (red solid curves Fig. 2(b,c)), as well as their interplay with reflectivity and transmissivity. This correlation is shown more clearly in Fig. 3, which plots measured maximum proton energy *E*_*p,max*_ including data from all target thicknesses versus measured absorptivity *η*_*L*_. *E*_*p,max*_ increases with increasing *η*_*L*_ and can be explained in the following picture. Assuming the acceleration is dominated by plasma expansion, we consider a simplified model that the magnitude of the plasma expansion velocity *v*_*exp*_ is related to the proton sound velocity 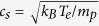, where *m*_*p*_ is the proton rest mass. Based on the absorptivity the number of hot electrons is estimated to be *N*_*e*_ = *η*_*L*_*E*_*L*_/*k*_*B*_*T*_*e*_ where *E*_*L*_ is the laser pulse energy and *k*_*B*_*T*_*e*_ is the average kinetic energy of electrons. Thus the resulting maximum proton energy is given as 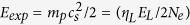, presenting a linear scaling with laser absorptivity *η*_*L*_ which agrees well with the observed increase trend as displayed in Fig. 3 and published models^12^. Also it becomes apparent that, when extrapolating our trend to *η*_*L*_ = 0, our experimental results suggest non-vanishing proton energy of ~3 MeV, while one would expect *E*_*exp*_ is reduced to zero as *η*_*L*_ = 0 with the simple scaling.

We are aware that the physical picture of plasma expansion will likely be more complicated, for example, due to the presence of two electron temperature distribution, multiple ion species, and combinations of both, etc, as described in refs 25, 26, 27, 28, 29, 30, 31. However, those complexities typically result in modulated spectral distributions, the maximum proton energy will remain dominated by the hot electrons^27^,^30^, in particular, the average kinetic energy will be zero for *η*_*L*_ = 0.

We propose to resolve the observed discrepancy with our experimental data by considering an additional velocity contribution from Light-sail form of radiation pressure acceleration (RPA-LS) in the following simple way. Independent of details of the laser pulse interaction with the foil-target, we simply assume that the radiation pressure accelerates a plasma bunch of total mass, *M* (which includes all particles of all ion species) to a single final energy. In addition to the laser pulse energy and bunch mass dependence, the energy of a proton within the bunch, given as 
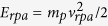
 is also determined by reflectivity and transmissivity (the number of reflected and transmitted photons respectively) where *v*_*rpa*_ = (1 + *r*_*L*_ − *t*_*L*_)*E*_*L*_/*Mc*^8^. We suggest that total proton energy measured in our experiments *E*_*p,max*_ results from the superposition of this centre of mass motion with plasma expansion, thus combining *E*_*rpa*_ and *E*_*exp*_ as follows,


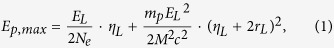


In a best fit of experimental data with Eq. 1 prediction both the electron number *N*_*e*_ and the total mass *M* are fitting parameters. Relying on the experimentally determined values for *η*_*L*_ and *r*_*L*_, and using *E*_*L*_ = 0.4 *J*, we obtain the solid black circles in Fig. 3, in reasonable agreement with the data, for *N*_*e*_ = 1.3 × 10^11^ and *M* = 1.28 × 10^−16^ *kg*. The contribution from plasma expansion alone is indicated in the black line in Fig. 3 with the characteristic linear dependence *E*_*exp*_ ∝ *η*_*L*_. The large contribution of plasma expansion is significant in the proton acceleration process; especially for higher absorptivity.

From these best fit results the RPA-LS contribution are plotted as the open red circles in Fig. 3. An upper and lower limits bracket the values of *E*_*rpa*_, corresponding respectively to *t*_*L*_ = 0 and *r*_*L*_ = 0. The upper limit imposes a quadratic decreasing scaling according to *E*_*rpa*_ ∝ (2 − *η*_*L*_)^2^ while the lower limit imposes a quadratic increasing scaling according to 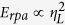. For a given *η*_*L*_ value, it is clear that the radiation pressure contribution is determined in part by *r*_*L*_ and *t*_*L*_, the target positional variation of which has been shown in Fig. 2(b). In the upper limit (*t*_*L*_ = 0 case) we determine a significant radiation pressure contribution 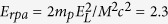 MeV for *η*_*L*_ = 0 (corresponding to the ideal case for RPA-LS where *r*_*L*_ = 1). Consequently, the observed correlation of proton energies with laser absorptivity is well reproduced by the coexistence of plasma expansion and RPA-LS mechanisms.

The upper and lower limits to RPA-LS energy contribution are only equal for *η*_*L*_ = 1, because only then reflectivity and transmissivity are uniquely defined as zero. At any fixed value of absorptivity *η*_*L*_, the width of the band that separates upper and lower limits scales as 1 − *η*_*L*_ where transmissivity *t*_*L*_ decreases from 1 − *η*_*L*_ at the lower limit to 0 at the upper limit with reflectivity *r*_*L*_ = 1 − *η*_*L*_ − *t*_*L*_.

It is also noteworthy that the best fit values for electron number, *N*_*e*_ and plasma bunch mass, *M* closely resemble those estimated from number of particles (ions and electrons) contained in the bulk of the target within the laser irradiated area. The DLC foils contain 90% carbon and 10% hydrogen which represents a total mass *M* = 1.3 × 10^−16^ *kg*) corresponds to about 0.6 × 10^10^ carbon ions, and a hot electrons number, *N*_*e*_ ≈ 10^11^.

In conclusion, by measuring the absorptivity and reflectivity of laser pulses interacting with nanometer thin foils, we show the concerted contributions of plasma expansion and radiation pressure acceleration to proton acceleration. Our simple explanation contributes to the understanding of the underlying physics of laser-driven ion acceleration. Further optimization would benefit from a deepened understanding of the momentum distribution of the electrons that remain bound to the target under different interaction conditions, by realistic simulations and experimental observation.

## Methods

The experiment was conducted with the ATLAS laser system at the Max-Planck-Institute for Quantum Optics in Garching; a table-top multi-TW Ti:Sapphire laser of central wavelength, 800 nm. The laser system delivers a linearly polarized 400 millijoule pulse downstream from a double plasma mirror (DPM) system. The pulse duration (full-width at half-maximum, FWHM) is 30 fs. As indicated in the experimental setup sketch of Fig. 1, a 90° off-axis parabolic (OAP) mirror is used to focus the pulses to a minimum spot size (FWHM) of 3 *μm*, yielding a peak intensity of 8 × 10^19^ W/cm^2^. The ATLAS laser pulse is normally incident on nm-thin DLC foils with thickness of 5–20 nm. A magnetic spectrometer with a long entrance slit is employed to measure the energy and angular distributions of the protons emitted in the direction of laser propagation^32^. With an Al foil of 45 µm thickness placed in front of Image plate (IP) ion detector, protons with kinetic energies above 2 MeV are recorded. We also note that carbon ions of energy above 43 MeV were not observed in the experiments.

The reflected laser light within the narrow incident cone was re-collimated by the OAP and delivered with two beam line mirrors with dielectric coating specified in the wavelength range of 740–860 nm, and later transported through a specially designed transmissivity (1%) fused silica-based mirror with full aperture (see Fig. 1). A large aperture lens then focusses this reflected light through a Fabry-Perot etalon and onto another CCD camera (14-bit). The etalon is a parallel mirror pair of 1 mm spacing that produces multiple beam images. The etalon guarantees that at least one image replica is adequately exposed (without being saturated), providing more reliable evaluation with much enhanced dynamic range as compared to a single camera image. The images are filtered with neutral density (ND) filters with optical density (OD) up to 4 and one long-pass filter (RG630). Along with the whole optical system, the setup allows a sufficient discrimination of the primary laser which we are interested in from secondary radiation such as harmonics. For example, the detection sensitivity of the fundamental laser is 6 orders of magnitudes higher than 2ω harmonic. The transmitted laser profile from a scatter screen located in front of the magnetic spectrometer entrance slit was imaged onto a CCD camera. Again, the picture is filtered with ND filters (OD 4) to discriminate the primary laser from the secondary radiation. Their detection sensitivity at the fundamental laser wavelength (800 nm), is 40 times larger compared to 2ω harmonic (400 nm).

The transmissivity is obtained by integrating the recorded laser profiles and compared to a reference shot with 100% transmissivity, i.e., a transmitted image without target (see the inset of Fig. 1). Analogously, the reflectivity is obtained from image integration. A calorimeter was used to cross-calibrate the absolute reflected energy to the integrated, recorded reflected laser images, where the total propagating efficiency of the whole optical beam path was characterised with a diode laser with 800 nm wavelength.

## Additional Information

**How to cite this article**: Bin, J. H. *et al*. Dynamics of laser-driven proton acceleration exhibited by measured laser absorptivity and reflectivity. *Sci. Rep.* **7**, 43548; doi: 10.1038/srep43548 (2017).

**Publisher's note:** Springer Nature remains neutral with regard to jurisdictional claims in published maps and institutional affiliations.
